# Effects of an *Armillaria mellea* Polysaccharide on Learning and Memory of D-Galactose-Induced Aging Mice

**DOI:** 10.3389/fphar.2022.919920

**Published:** 2022-07-18

**Authors:** Hongyu Li, Guangyu Xu, Guangxin Yuan

**Affiliations:** ^1^ School of Pharmacy, Beihua University, Jilin, China; ^2^ School of Basic Medicine, Jiamusi University, Jiamusi, China

**Keywords:** *Armillaria mellea* polysaccharide, learning and memory, antioxidant, hippocampus, nerve cells

## Abstract

*Arm* *illaria mellea* has been known and used in traditional medicine in East Asia for hundreds of years. It has already been reported that *A. mellea* extracts have various pharmacological effects, and the polysaccharides of *A. mellea* exhibit antioxidant and anti-apoptotic activities. In this study, a water-soluble polysaccharide (AMP-N-a-1), with an average molecular weight of 17 kD, was isolated and purified from the water extract of *A. mellea* using DEAE-52, Sepharose CL-4B, and Sephadex G-100 column chromatography. AMP-N-a-1 was mainly composed of Man (1.65%), Glca (1.64%), Rha (1.82%), Gala (2.49%), Glc (90.48%), Gal (0.89%), Xyl (0.42%), and Ara (0.61%). AMP-N-a-1 was used to study the effect on the learning and memory of mice and its underlying mechanisms. The results showed that AMP-N-a-1 could significantly increase the activities of catalase (CAT) and superoxide dismutase (SOD) and reduce the content of nitric oxide (NO) in mouse brain tissue. Meanwhile, AMP-N-a-1 could reduce the contents of norepinephrine (NE) and dopamine (DA) but could increase the content of 5-hydroxytryptamine (5-HT) in mouse brain tissue. In addition, the immunofluorescence experiment showed that AMP-N-a-1 could promote the proliferation of hippocampal dentate gyrus neurons. The above results indicate that AMP-N-a-1 can significantly improve the learning and memory of mice, and the mechanism may be that AMP-N-a-1 can participate in the regulation of learning and memory through a variety of ways.

## Introduction

Learning and memory are important functions of the brain and an advanced neurophysiological activity of mammals, but the ability of learning and memory shows a downward trend with aging. With the improvement of living conditions and the increase in the proportion of the elderly population, there will be more and more people who are perplexed by the declined learning and memory abilities. Therefore, the early prevention and treatment of the symptoms is one of the hot spots, with a very broad market prospect. The hippocampal structure is closely related to the ability of learning and memory, since many studies have shown that the neurogenesis of the hippocampal dentate gyrus can affect the performance of spatial learning and memory in aged mice (Kempermann and Gage, 2002), and the long-term potentiation (LTP) formation of the dentate gyrus (Praag et al., 2005), demonstrated that the hippocampal neurogenesis should be closely related to the spatial learning function. Snyder et al. (2005) found that the defective neurogenesis in the dentate gyrus could cause learning and memory disorders, and inhibiting the number of new neurons in the dentate gyrus using low-dose radiation could affect the induction of LTP and lead to the decline in learning and memory. Meltzer et al. (2005) reported that the regenerative neurons in the hippocampal dentate gyrus could enhance the body’s learning ability. It was further confirmed by Kempermann et al. (2004) that the dentate gyrus of the hippocampus had the function of transient information storage and the obtained information could be re-encoded and used to participate in the transmission process of neural synapses in the CA3 region, and this process was related to the proliferation of granular layer neurons in the dentate gyrus. These studies suggest that the neurogenesis in the hippocampal dentate gyrus should be closely associated with the ability of learning and memory. Therefore, it is of great significance to study the effects of drugs on the neurogenesis in the hippocampal dentate gyrus and thereby develop drugs that can prevent and improve the deterioration of learning and memory.

D-galactose can be converted into glucose to participate in the metabolic process in the body, but an excessive dose of D-galactose can cause metabolic disorders and induce oxidative stress reaction in the body and the apoptosis of neurons, which is similar to the natural aging process. Therefore, the D-galactose model has become a commonly used model for the study on the cognitive impairment of aging (Sadigh-Eteghad et al., 2017). Many studies have shown that the learning and memory abilities of animals are closely related to the monoamine neurotransmitters in the central nervous system. Aging can cause changes in monoamine neurotransmitters, such as norepinephrine (NE), dopamine (DA), and 5-hydroxytryptamine (5-HT), and the changes are directly related to the decline in learning and memory. In general, in the central nervous system, the secretion of various monoamine neurotransmitters is maintained at a certain level, and their contents are balanced to maintain the normal function of the nervous system. In aging, a large number of neuronal cells are apoptotic, learning and memory decline, and the metabolism of monoamine neurotransmitters in the brain is disordered, thereby inducing changes in their contents (Fuente et al., 2003; Braskie et al., 2011; Chalermpalanupap et al., 2013; Oliveira et al., 2010).

*Armillaria mellea* (Vahl) P. Kumm, also known as the hazelnut or honey mushroom, has been used as a medically beneficial edible mushroom in traditional Chinese medicine. The medical effect of *Armillaria mellea* was originally included in the ancient Chinese medicine classics “Shennong’s Herbal Classic” and “Compendium of Materia Medica” (Lin et al., 2021), and it is recorded that *Armillaria mellea* could be used in epilepsy, limb numbness, and headache in traditional Chinese medicine (Compilation Board of National Chinese Herbal Medicine Collection, 1996; Nanjing University of Traditional Chinese Medicine, 2006). It has been widely used in the treatment of neurological diseases, such as migraines, headaches, neurasthenia, insomnia, and epilepsy (Yang et al., 1984). In recent years, it has been reported that the extracts from *Armillaria mellea* show various pharmacological effects, including retarding aging, hypnosis, calmness, improving immunity, and neuron protection (Chen et al., 2013; Wang et al., 2016; Yi, 2020; Li et al., 2021). Studies have shown that *Armillaria mellea* polysaccharide (AMP) can treat Alzheimer’s disease (AD) through anti-apoptosis and anti-oxidation (Bozena et al., 2011). However, systematic studies on the relationship between the effect of AMP on the neurogenesis of the hippocampus and the improvement of learning and memory are still insufficient. In this study, a mouse learning and memory impairment model was established through the administration of D-galactose first, and then, AMP was intragastrically given to the mice to observe the effects of AMP on the learning and memory ability and the proliferation of hippocampus nerve cells and explore the mechanisms through which AMP can prevent and improve learning and memory impairment.

## Materials and Methods

### Materials and Reagents

The dried fruiting bodies of *Armillaria mellea* (Vahl) P. Kumm. were purchased from Antu Xinyu Edible and Medicinal Fungus Research and Development Co., Ltd. (Yanbian, China). *Armillaria mellea* colonies were artificially cultivated in the planting base of the company. The fresh fruiting bodies were collected from July to August every year, cleaned with water, and dried in the sun. In addition, 90 male Kunming mice, 4–5 weeks old, and weighing 15 ± 2 g, were purchased from Experimental Animal Center, Jilin University, and the mice were raised in separate cages at 22–25°C in a light-dark cycle of 12 h, with access to food and water *ad libitum*. Superoxide dismutase (SOD) and catalase (CAT) kits were purchased from the Nanjing Jiancheng Bioengineering Institute (China); the nitric oxide (NO) kit was from Beyotime Biotechnology Company (China); NE, DA hydrochloride, 5-HT hydrochloride, and bromodeoxyuridine (BrdU) markers were obtained from Sigma-Aldrich, United States; all monoclonal antibodies and the immunofluorescence secondary antibodies were purchased from Abcam, England; and the high-performance liquid chromatography (HPLC) grade methanol and trisodium citrate were delivered by Merck, Germany. Other reagents and deionized water were of analytical grade.

### Extraction, Separation, and Purification of a Polysaccharide From *Armillaria mellea* Fruiting Bodies

The fruiting bodies were first cleaned, with the impurities removed from the surface, then washed, and finally dried and cut into small pieces. Then, 100 g of the dried fungal pieces was extracted twice with 1,000 mL of 90% ethanol. The extracts were mixed, the mixture was filtered using a Brinell funnel, and the residue was dried at 45°C at atmospheric pressure. The residue was extracted three times with 20 times the volume (V/W) of distilled water at 90°C, 1 h each time. The extracts were filtered, and the filtrates were mixed. The mixture was concentrated, then the concentrated solution was mixed with 95% ethanol five times its volume, and the solution was left standing at 4°C for 24 h for its precipitation. The precipitate was collected by centrifugation and dissolved in distilled water, and the protein in it was removed using the Sevag method (Song et al., 2019). The precipitate in which the protein had been removed was dialyzed with distilled water for 48 h, concentrated under reduced pressure, freeze dried, and then cleaned with absolute ethanol, acetone, and ether. Finally, a mixture of polysaccharides named AMPs was obtained, with a yield of 6.24%.

The AMPs were dissolved in distilled water. The solution was centrifuged (4,000 rpm for 10 min, 5418R Eppendorf, Germany), and then, the supernatant was loaded into a DEAE-52 column (1.5 cm × 60 cm) and eluted with distilled water, 0.2 M NaCl, and 0.5 M NaCl solutions at a flow rate of 1 mL min^−1^ each. The content of polysaccharides in the eluent was tracked using the phenol–sulfuric acid method to obtain the neutral sugar N fraction (distilled water), acidic sugar A fraction (0.2 M NaCl), and acidic polysaccharide B fraction (0.5 M NaCl). The component AMP-N (the neutral fraction of AMPs) obtained using elution with distilled water was chromatographed on a Sepharose CL-4B column (2.6 cm × 100 cm) for further separation and purification, in which the eluent was 0.15 M NaCl solution and the elution flow rate was 0.4 mL min^−1^, and finally, two components were obtained. After dialysis and freeze drying, the two components were named AMP-N-a and AMP-N-b. AMP-N-a was further purified on a Sephadex G-100 column, in which the eluent was distilled water and the flow rate was 0.4 mL min^−1^. The main polysaccharide components were named AMP-N-a-1 after combination, dialysis, and freeze drying, and the final yield was 1.23%. The above process was repeated until the amount of AMP-N-a-1 required for subsequent experiments was obtained.

### Molecular Weight Determination

An appropriate amount of AMP-N-a-1 was dissolved in 0.2 M NaCl solution to prepare a sample solution at a concentration of 5 mg mL^−1^. The solution was filtered through a 0.22 μM filtration membrane (Millex-LG hydrophilic PTFE membrane, Millipore, United States), and then, the residue was analyzed using high-performance gel permeation chromatography (HPGPC), in which an LC-20AT system of Shimadzu company, a RID-20A differential refractive detector, and a TSK-gel G-4000 PW_XL_ chromatographic column (7.8 mm × 300 mm) were used. The column temperature was 40°C, 0.2 M NaCl was used as a mobile phase, the flow rate was 0.6 mL min^−1^, and the injection volume was 20 μL. The dextran with molecular weights of 1, 5, 12, 25, and 50 kDa was used for calibration to draw the standard curve.

### Monosaccharide Composition Analysis

An appropriate amount of AMP-N-a-1 weighed accurately was dissolved in 2 M hydrochloric acid–methanol solution to prepare a 2 mg mL^−1^ sample solution. The solution was filled with N_2_ (N-EVAP-12, Organomation, United States) and then placed in a constant temperature metal bath at 80°C for 16 h for hydrolysis. The reaction sample solution was blow dried using a nitrogen blowing instrument at 120°C for 1 h for removing the methanol–acetic acid, then added with trifluoroacetic acid for 1 h and blow-dried again. The hydrolysate was precolumn derivatized with 1-phenyl-3-methyl-5-pyrazolone (PMP), filtered through a 0.22 μM filter membrane (Millex-LG hydrophilic PTFE membrane, Millipore, United States), and then detected using a Shimadzu HPLC system (LC-20AT pump and SPD-20A UV-Vis Detector) and COSMOSIL 5C18-PAQ chromatographic column (4.6 mm × 250 mm). The monosaccharide composition of AMP-N-a-1 was identified, and their contents were calculated based on the retention time and peak area of the standard monosaccharide.

### Animal Grouping and Administration

A total of 90 male Kunming mice were randomly divided into five groups, with 18 mice in each group: the control group, the model group, and the AMP-N-a-1 groups (0.025, 0.05, and 0.1 g kg^−1^·d^−1^).

Mice in the model group and AMP-N-a-1 groups were intraperitoneally injected with D-galactose (200 mg kg^−1^·d^−1^), and those in the control group were given saline (20 mL kg^−1^·d^−1^) in the same way continuously for 42 days to establish a mouse learning and memory impairment model (Yu et al., 2007). Mice in the AMP-N-a-1 groups were given different doses of AMP-N-a-1 by gavage for 14 consecutive days from the 29th day, and mice in the control and model groups were given an equal volume of saline in the same way.

### Morris Water Maze Test

According to the methods described in the literature (Gandhi et al., 2000; Nam et al., 2019), the Morris water maze test for the observation of the ability of learning and memory of mice was performed after the model was established. The test lasted for 5 days, in which the first 4 days were the period of training adaptation. The mice were trained four times daily, and the training interval was 120 s. The mice were put into the water from four different quadrants. A platform was set up in the water, the water surface was 2 cm higher than the platform, and the water temperature was 22.0 ± 0.5°C. The number and latency of mice that reached the platform were observed and recorded. If a mouse did not find the platform within 120 s, it should be led to the platform, and the latency was recorded as 120 s. On the fifth day, the platform was removed, and the site where the mice were put into the water was randomly selected for the formal testing. The locus of the mice looking for the platform area was recorded within 120 s, including the residence time in the effective area, the number of mice crossing the platform area, and the latency of mice reaching the platform area, which were taken as the test results of behavioral experiments.

### Detection of Oxidative Stress-Related Enzymes

After the end of the Morris water maze test, half of the mice in each group were decapitated, and their whole brain tissue samples were kept on ice. The whole brain tissues of mice were weighed, added to a phosphoric acid buffer solution, homogenized in an ice bath, and centrifuged at 4°C and 14,000 rpm for 20 min to obtain the supernatants. Then, SOD activities, NO contents, and CAT activities in the supernatants were determined using the xanthine oxidase method, chemiluminescence method, and spectrophotometry, respectively, according to the instructions of the kits.

### Detection of Monoamine Transmitters in the Mice’s Brain Tissues

The brain homogenate prepared above was centrifuged at 4°C and 14,000 rpm for 20 min to obtain the supernatants. The supernatants were filtered using a 0.45 μM filter membrane (Millipore, United States), and 100 μL of the filtrates was injected into a high-performance liquid chromatograph for the detection of monoamine transmitters. Conditions of the high-performance liquid chromatograph (E2695, Waters, United States) for the detection were as follows: mobile phase: trisodium citrate (20 mmol L^−1^), pH 4.50 adjusted with hydrochloride, 0.45 μM filter membrane, 5% (V/V) methanol, and ultrasonic degassing; flow rate: 1 mL min^−1^; column temperature: 5°C; fluorescence detector wavelength: excitation wavelength at 280 nm and emission wavelength at 315 nm; and peak area quantification.

### Preparation of Brain Tissue

After the end of the Morris water maze test, half of the mice in each group were given BrdU (50 mg kg^−1^) via intraperitoneal injection three times a day. After the continuously intraperitoneal injection of BrdU for 5 days, the mice were anesthetized with 10% chloral hydrate (300 mg kg^−1^) intraperitoneally. The anesthetized mice’s hearts were rapidly perfused with normal saline first and then slowly with 4% paraformaldehyde for 30 min, and when it was found that their whiskers moved, front and hind legs twitched, and bodies became rigid, they were quickly decapitated to take their brains. The brain tissues were fixed in 4% paraformaldehyde for 6 h, deposited with 30% sucrose solution, and embedded with optimal cutting temperature compound; the embedded tissues were placed in a liquid nitrogen sink for several minutes and then immediately kept in a −80°C refrigerator. The brain tissue sections were prepared using a cryostat microtome. Based on the mice’s brain stereotaxic atlas, the continuous coronary hippocampus slices with a thickness of 20 μM were prepared (Pouzet et al., 2002).

### Histopathological Observation of Hippocampus HE Staining

The sections were taken out from the refrigerator (−20°C) and placed at room temperature for 20 min until the tissue was dry. Samples were stained using the standard protocols for hematoxylin and eosin staining (Paxinos and Franklin, 2003). At first, the sections were stained using Mayer’s hematoxylin solution for 2–5 min and washed briefly using running tap water for over 25 min. After being rinsed in distilled water, the sections were stained using eosin solution for 5 min and rinsed in distilled water for a few seconds to remove the excess eosin. Then, the sections were deparaffinized and dehydrated using 70, 80, 90, and 100% ethanol and mounted with polyvinyl alcohol (PVA) after being cleaned in xylene three times. After staining, the samples were observed under a BX51 microscope with 400-fold magnification (Olympus, Japan).

### Double Immunofluorescence Staining

The positive cells were stained using double immunofluorescence staining of BrdU and neuronal nuclei (NeuN). The slices were washed with phosphate-buffered saline (PBS) and then put in citrate buffer at pH 6.0, which was heated using a microwave for 5 min for the antigen repair. PBS containing 0.3% Triton X was added to the slices. Five minutes later, the slices were incubated with the blocking buffer 10% goat serum for 1 h, and then, the blocking buffer was discarded, rat BrdU monoclonal antibody (1:200) and mouse NeuN monoclonal antibody (1:500) were added to them, and they were kept at 4°C overnight. After the slices were washed with PBS for 5 min, which was repeated three times, they were incubated with the goat anti-mouse IgG (1:1,000) labeled with Alexa Fluor 488 and that (1:4,000) labeled with AlexaFluor 594 at room temperature for 3 h. The slices were washed with PBS for 5 min, which was repeated three times, and were then mounted with PVA solution.

After the mounting, the images were taken under a fluorescence microscope with 200-fold magnification (Olympus, Japan). The expression of NeuN detected by the fluorescence staining was observed under the green light excited by the 488 nm wavelength, and the expression of BrdU was examined under the red light excited by the 594 nm wavelength using a fluorescence microscope. The 10 views with the strongest fluorescence expression in each section were selected and then photographed and preserved for manual counting. BrdU- and NeuN-stained cells in the 10 views were randomly counted manually, the averages were calculated, and the ratios of BrdU^+^/NeuN^+^ cells were calculated (Gilbertson, 2012).

### Statistical Analysis

The one-way analysis of variance was carried out using SPSS 12.0 statistical software. The data were expressed by mean ± standard deviation (mean ± s), and *p* < 0.05 was considered a difference with statistical significance.

## Results

### Preparation of AMP-N-a-1

The crude polysaccharide AMPs were obtained from *Armillaria mellea* by water extraction and alcohol precipitation, with a yield of 4.16%. AMPs were gradient eluted by DEAE-52 column chromatography to obtain a neutral sugar fraction AMP-N (70.2%) and acidic sugar fractions AMP-a (4.3%) and AMP-b (0.3%). Fraction AMP-N was further purified by Sepharose CL-4B, and two obvious elution peaks were obtained. The two fractions were collected, lyophilized, and named AMP-N-a (with a yield of 49%) and AMP-N-b (with a yield of 11.3%). Fraction AMP-N-a was further purified by Sephadex G-100 to obtain a fraction with a uniform molecular weight, and the fraction was named AMP-N-a-1, with a yield of 57.28%.

### Monosaccharide Composition and Molecular Weight

**FIGURE 1:**
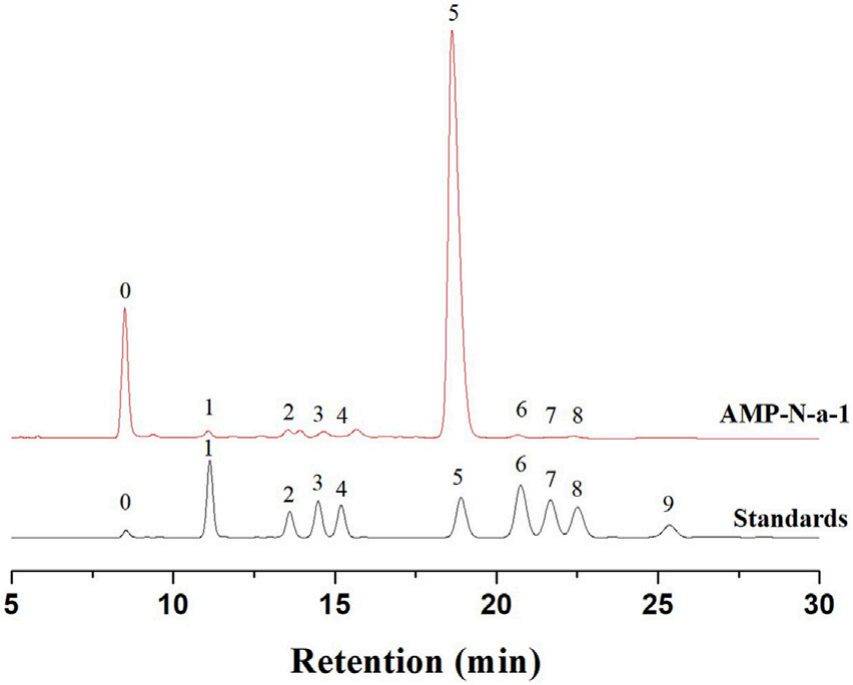
Chromatograms of nine PMP-derivative monosaccharide standards (0-PMP, 1-Man, 2-Glca, 3-Rha, 4-Gala, 5-Glc, 6-Gal, 7-Xyl, 8-Ara, and 9-Fuc) and AMP-N-a-1.

The molecular weight of AMP-N-a-1 was determined using HPGPC, and the results showed that its molecular weight was approximately 17 kD. The HPLC analysis showed that AMP-N-a-1 was mainly composed of Man (1.65%), Glca (1.64%), Rha (1.82%), Gala (2.49%), Glc (90.48%), Gal (0.89%), Xyl (0.42%), and Ara (0.61%) (Figure 1).

### Effects of AMP-N-a-1 on the Mice’s Performance in Morris Water Maze Test

The results showed that compared with those in the control group, the residence time of mice in the effective zone was significantly shortened, the times of mice crossing the platform was significantly reduced, and the latency to reach the platform was significantly prolonged in the model group (*p* < 0.05 or *p* < 0.01). In addition, compared with those in the model group, the residence time of mice in the effective zone was significantly prolonged, the number of mice crossing the platform was significantly increased, and the latency to reach the platform was significantly shortened in the 0.05 and 0.1 g kg^−1^ AMP-N-a-1 groups (*p* < 0.05 or *p* < 0.01), but the three indices in the 0.025 g kg^−1^ dose group were not significantly different. The results are shown in Table 1.

**TABLE 1:** Effects of AMP-N-a-1 on the performance of mice with learning and memory disorders in the Morris water maze test (*n* = 18, mean ± s).

Group	Dose/g·kg^−1^	Effective zone residence time/s	Number of crossing platform/times	Latency/s
Control		1.16 ± 0.81	2.52 ± 1.26	33.82 ± 2.94
Model		0.64 ± 0.39^*^	0.92 ± 0.37^**^	50.31 ± 4.25^**^
AMP-N-a-1	0.025	0.90 ± 0.27	1.28 ± 0.55	46.89 ± 4.82
	0.05	1.05 ± 0.48^#^	1.98 ± 0.91^#^	41.06 ± 4.67^#^
	0.1	1.09 ± 0.55^#^	2.36 ± 1.42^##^	39.75 ± 2.94^##^

Compared with the control group, *p < 0.05, **p < 0.01.

Compared with the model group. ^#^ *p* < 0.05, ^##^ *p* < 0.01.

### Regulation of AMP-N-a-1 on Oxidative Stress-Related Enzymes in Mice

SOD and CAT activities and NO contents in the brain tissue of mice with learning and memory impairment were detected after the behavioral experiments. The results showed that the CAT and SOD activities in the brain tissue of mice in the model group were lower than those in the control group (*p* < 0.01) and the NO contents were higher than those in the control group (*p* < 0.01). The effects of different doses of AMP-N-a-1 on CAT and SOD activities and NO contents were different, in which 0.05 and 0.1 g kg^−1^ AMP-N-a-1 could increase the activity of CAT in the mice’s brain tissue statistically significantly compared with those in the model group (*p* < 0.01), but the change in the activity of CAT in the 0.025 g kg^−1^ AMP-N-a-1 group was not significantly different from that in the model group. The three doses of AMP-N-a-1 could increase the activity of SOD and decrease the content of NO in the mouse brain tissue (*p* < 0.01). The results are shown in Table 2.

**TABLE 2:** Effects of AMP-N-a-1 on the oxidative stress–related enzymes in the brain tissue of mice with learning and memory disorders (*n* = 18, mean ± s).

Group	Dose/g·kg^−1^	CAT/U·mg (prot)^−1^	SOD/U·mg (prot)^−1^	NO/μmol·L^−1^
Control		23.38 ± 6.36	276.76 ± 65.61	6.74 ± 3.66
Model		10.25 ± 2.28^**^	155.95 ± 26.89^**^	32.85 ± 9.91^**^
AMP-N-a-1	0.025	16.56 ± 3.12	216.69 ± 40.62^##^	15.02 ± 5.33^##^
	0.05	24.39 ± 7.31^##^	236.38 ± 5.32^##^	11.97 ± 1.73^##^
	0.1	27.28 ± 6.75^##^	261.83 ± 59.21^##^	10.94 ± 4.22^##^

Compared with the control group, **p < 0.01.

Compared with the model group,^#^ *p* < 0.05, ^##^ *p* < 0.01.

### Effects of AMP-N-a-1 on Monoamine Transmitters in the Mice Brain Tissues

The content of various monoamine neurotransmitters in the brain tissue is of great significance for the treatment and diagnosis of memory impairment. As shown in Table 3, compared with those in the blank control group, the content of NE and DA in the brain tissue of mice in the model group increased and the content of 5-HT significantly decreased (*p* < 0.05). In addition, compared with those in the model group, the content of NE and DA in the 0.05 and 0.1 g kg^−1^ AMP-N-a-1 groups decreased significantly (*p* < 0.05, *p* < 0.01), and the content of 5-HT in the 0.025, 0.05, and 0.1 g kg^−1^ AMP-N-a-1 groups increased significantly (*p* < 0.05), thereby suggesting that AMP-N-a-1 can regulate the learning and memory impairment by reducing the content of NE and DA and increasing the content of 5-HT in the brain tissue of mice.

**TABLE 3:** Effects of AMP-N-a-1 on NE, DA, and 5-HT contents in the brain tissue of mice with learning and memory disorders (*n* = 18, mean ± s).

Group	Dose/g·kg^−1^	NE/ng·g^−1^	DA/ng·g^−1^	5-HT/ng·g^−1^
Control		5.45 ± 1.32	1.27 ± 0.41	1.82 ± 0.36
Model		7.28 ± 1.21^*^	1.57 ± 0.35^*^	0.71 ± 0.39^*^
AMP-N-a-1	0.025	6.56 ± 0.98	1.37 ± 0.26	1.39 ± 0.36^#^
	0.05	5.52 ± 1.57^#^	1.16 ± 0.26^#^	1.57 ± 0.45^#^
	0.1	4.43 ± 0.92^##^	1.17 ± 0.22^#^	1.92 ± 0.48^##^

Compared with the control group, *p < 0.05.

Compared with the model group,^#^ *p* < 0.05, ^##^ *p* < 0.01.

### Pathological Observation in Hippocampus of Mice

**FIGURE 2:**
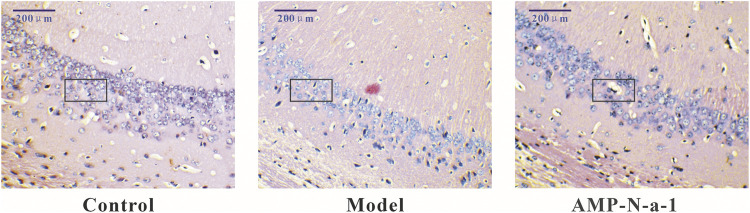
HE staining of neurons in the hippocampal CA3 region in the control group, model group, and 0.1 g kg^−1^ AMP-N-a-1 group.

As shown in Figure 2, the neurons in the hippocampal CA3 region of control mice were clear and intact. The neurons in the same region of the model mice were severely damaged, the cells were irregular, the cell vacuolization was increased, and the intercellular space became enlarged. The number of abnormal and missing neurons in the hippocampal CA3 region decreased in the 0.1 g kg^−1^ AMP-N-a-1 group, and the cells arranged closer than those in the model group (Table 4). The results indicate that the neurons in the hippocampus can be destroyed and lost by the intraperitoneal injection of D-galactose, but the neurons can be protected from damage by the intragastric administration of AMP-N-a-1.

**TABLE 4:** Effects of AMP-N-a-1 on the numerical density of neurons in the CA3 region of the hippocampus in mice with learning and memory disorder (*n* = 18, mean ± s).

Group	Numerical density of neurons/(× 10^−4^ μM^−3^)
Control	8.5 ± 0.6
Model	6.7 ± 0.8^**^
AMP-N-a-1 (0.1 g kg^−1^)	8.2 ± 0.8^##^

Compared with the control group, **p < 0.01.

Compared with the model group,^#^ *p* < 0.05, ^##^ *p* < 0.01.

### Effects of AMP-N-a-1 on the Proliferation of Neurons in the Hippocampal Dentate Gyrus of Mice with Learning and Memory Disorders

**FIGURE 3:**
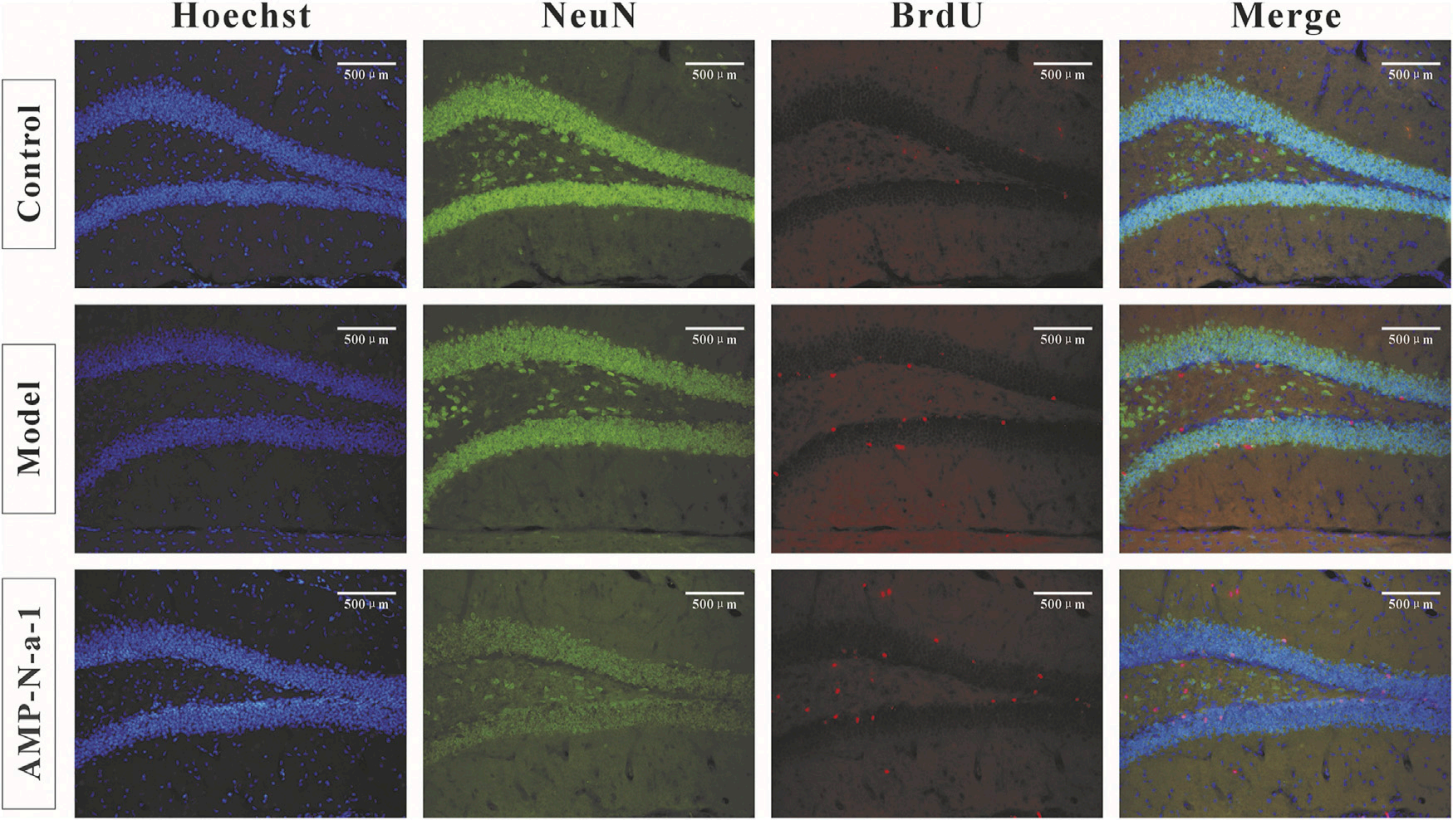
Double BrdU/NeuN expressions in the hippocampal dentate gyrus in the control group, model group, and 0.1 g kg^−1^ AMP-N-a-1 group.

**FIGURE 4:**
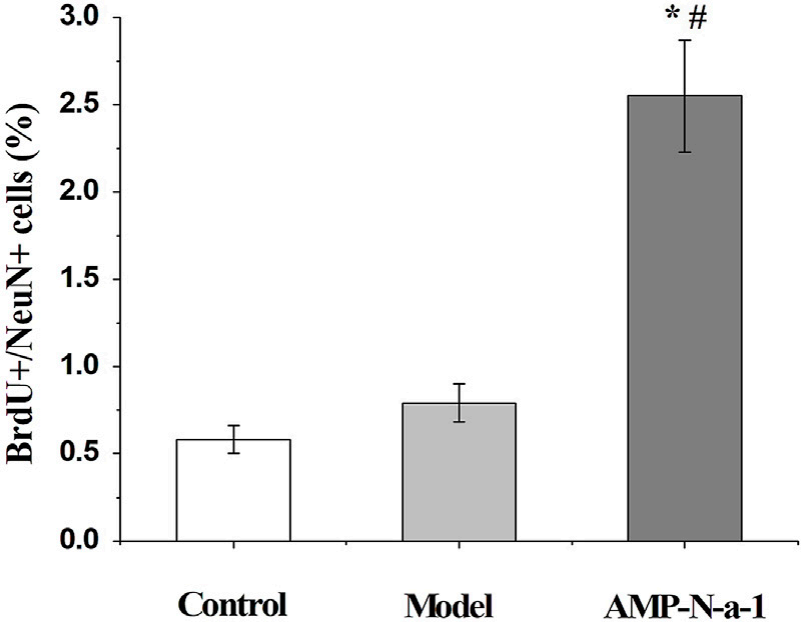
Ratios of BrdU^+^/NeuN^+^ cells in the control group, model group, and 0.1 g kg^−1^ AMP-N-a-1 group. Notes: compared with those in the control group, *p < 0.05; compared with those in the model group, ^#^ *p* < 0.05.

The newly proliferated neural cells were labeled with BrdU, and the nuclear antigen of the mature neural cells was labeled with Anti-NeuN. The immunofluorescence results are shown in Figure 3. Because BrdU could be used to label the new neural precursor cells and there might be individual differences of the neural cells in the dentate gyrus of the hippocampus, the ratio of BrdU^+^/NeuN^+^ cells were applied to illustrate the cell proliferation in the dentate gyrus in order to eliminate the differences. As shown in Figure 4, the BrdU^+^/NeuN^+^ ratio in the model group was slightly higher than that in the blank control group (*p* > 0.05), and the BrdU^+^/NeuN^+^ ratio in the 0.1 g kg^−1^ AMP-N-a-1 group was significantly higher than that in the model group and the blank control group (*p* < 0.05), thereby indicating that the cell proliferation may increase slightly after the intraperitoneal injection of D-galactose in mice, and the increase in BrdU^+^ cells may be a compensatory increase induced by the body’s repair of subacute brain injury. The administration of AMP-N-a-1 by gavage can significantly promote the proliferation of hippocampal neurons in the dentate gyrus, which may be involved in the improvement of learning and memory impairment in mice.

## Discussion

In this study, the Morris water maze system was applied to observe the effects of different doses of AMP-N-a-1 on the spatial learning and memory in mice. The Morris water maze test is a preferred classical method for the behavioral experiments of learning and memory. It is widely used in the applied research field of learning and memory, AD, and neurobiology (Fritschy et al., 1992; D'Hooge and De Deyn, 2001), primarily including the place navigation test and the spatial probe test. It is used to test how the experimental animals learn to look for a hidden platform at a fixed position in the course of repeated training and the animals’ stable spatial awareness established in their memory. The Morris water maze test can practically reflect the experimental animals’ learning and memory in terms of space and orientation. This study found that the intraperitoneal injection of D-galactose could significantly damage spatial learning ability in mice, which was consistent with that of the known research reports (Zhao et al., 2009). The results showed that the residence time in the effective zone and the number of mice crossing the platform were significantly reduced in the model group, and the latency of the mice’s reaching the platform was significantly prolonged compared with that in the control group (*p* < 0.05 or *p* < 0.01). In addition, compared with those in the model group, the spatial learning and memory were significantly enhanced, and the above indices were significantly improved in mice treated with the intragastric administration of AMP-N-a-1, thereby demonstrating that AMP-N-a-1 can improve the learning and memory impairment induced by the intraperitoneal injection of D-galactose, with a therapeutic effect on the acquired learning and memory disorders.

AD is a central nervous system degenerative disease. The aging of cells is one of the most dangerous factors, and the incidence of AD is closely associated with the damage induced by free radicals (Markesbery, 1997; Luo et al., 2003; Dubinina & Pustygina, 2007; Perry et al., 2008). Based on the theory of free radical damage, the nerve tissue is more vulnerable to the attack of oxygen free radicals owing to its structure, which is distinct from that of the other tissues (Carney & Carney, 1994; Zhu et al., 2007; Massaad et al., 2009). A large number of free radicals produced in the process of the cell metabolism will damage macromolecules in the body, such as DNA, to accelerate the death of cells and cause degenerative diseases, which can cause the aging of the body and the brain (Pouzet et al., 2002). The results of this study showed that AMP-N-a-1 could significantly improve the activity of SOD and CAT in the brain tissue of mice and decrease the content of NO with statistical significance compared with those in the model group. Based on the results, it can be believed that regulating the activity of some oxidases and exerting the antioxidant effect may be one of the mechanisms through which AMP-N-a-1 can improve the learning and memory in the mice.

Monoamine neurotransmitters in the brain are a kind of neurotransmitter involved in the regulation of learning and memory, including NE, DA, and 5-HT. They are important substances for information transmission in the central nervous system. The content of various monoamine neurotransmitters in the brain tissue is of great significance for the treatment and diagnosis of memory impairment. Studies have shown that NE can facilitate the consolidation of the representation of the information of nervous system activities, and DA and 5-HT can also enhance memory. Birthelmer et al. (2003) have shown that the decrease of monoamine neurotransmitters in the brain can cause the loss of neurons, cell death and degeneration, and the impairment of learning and memory ability. In addition, it has been reported that NE and 5-HT can interact with the cholinergic system, and the noradrenergic nerve and serotoninergic system scan affect the cholinergic system by influencing astrocytes to have an impact on learning and memory together (Markesbery & Lovell, 2007). In this study, the effects of AMP-N-a-1 on the monoamine neurotransmitters were studied, which should be one of the important links in the pathogenesis of AD, in order to provide a more direct evidence for the improvement of AMP-N-a-1 on learning and memory. This study showed that AMP-N-a-1 could significantly reduce the content of NE and DA and increase the content of 5-HT in the brain tissue of mice with learning and memory disorders, thereby revealing that the effect of AMP-N-a-1 on learning and memory may be associated with reducing the content of NE and DA and increasing the content of 5-HT.

The structure of the hippocampus is closely related to learning and memory. It was found in this study that the intragastric administration of AMP-N-a-1 could protect nerve cells in the hippocampus and produce an anti-injury effect to improve the learning and memory impairment. The study by Hastings and Gould (2015) showed that the neurons of the hippocampal dentate gyrus could extend their axons to CA3 within 4–10 days, often be surrounded by synaptic vesicles, and be integrated into the neuronal circuits related to learning and memory in the hippocampus. This study found that the intragastric administration of AMP-N-a-1 could promote the cell proliferation in the hippocampal dentate gyrus, and the effect of AMP-N-a-1 on the mice’s learning memory in each group was consistent with its effect on the cell proliferation, thereby suggesting that the improvement of AMP-N-a-1 in learning and memory is related to promoting the cell proliferation of the hippocampal dentate gyrus.

## Conclusion

A mouse learning and memory impairment model was established by the intraperitoneal injection of D-galactose, and the mice with learning and memory impairment were intragastrically given AMP-N-a-1. The results indicate that AMP-N-a-1 can significantly improve the learning and memory of mice. This study reveals that AMP-N-a-1 may participate in the regulation of learning and memory through a variety of ways, such as regulating the activity of some oxidases, reducing the content of NE and DA in the brain tissue, increasing the content of 5-HT, and promoting the cell proliferation of the hippocampal dentate gyrus.

## Data Availability

The original contributions presented in the study are included in the article/supplementary material, and further inquiries can be directed to the corresponding author.
